# Linked randomised controlled trials of face-to-face and electronic brief intervention methods to prevent alcohol related harm in young people aged 14–17 years presenting to Emergency Departments (SIPS junior)

**DOI:** 10.1186/s12889-015-1679-4

**Published:** 2015-04-10

**Authors:** Paolo Deluca, Simon Coulton, M Fasihul Alam, David Cohen, Kim Donoghue, Eilish Gilvarry, Eileen Kaner, Ian Maconochie, Paul McArdle, Ruth McGovern, Dorothy Newbury-Birch, Robert Patton, Ceri Phillips, Thomas Phillips, Ian Russell, John Strang, Colin Drummond

**Affiliations:** Institute of Psychiatry, Psychology & Neuroscience, King’s College London, London, UK; Centre for Health Services Studies, University of Kent, Canterbury, UK; Swansea Centre for Health Economics, College of Human and Health Sciences, Swansea University, Swansea, Wales UK; Health Economics and Policy Research Unit, University of South Wales, Pontypridd, UK; Northumberland Tyne and Wear NHS Foundation Trust, Newcastle, UK; Institute of Health and Society, Newcastle University, Newcastle, UK; Paediatric Emergency Medicine, Imperial College, London, UK; School of Health and Social Care, Teesside University, Middlesbrough, UK; School of Psychology, University of Surrey, Guildford, UK; Humber NHS Foundation Trust, Willerby, UK; College of Medicine, Swansea University, Swansea, Wales UK

**Keywords:** Adolescents, SBI, eBI, Emergency Department

## Abstract

**Background:**

Alcohol is a major global threat to public health. Although the main burden of chronic alcohol-related disease is in adults, its foundations often lie in adolescence. Alcohol consumption and related harm increase steeply from the age of 12 until 20 years. Several trials focusing upon young people have reported significant positive effects of brief interventions on a range of alcohol consumption outcomes. A recent review of reviews also suggests that electronic brief interventions (eBIs) using internet and smartphone technologies may markedly reduce alcohol consumption compared with minimal or no intervention controls.

Interventions that target non-drinking youth are known to delay the onset of drinking behaviours. Web based alcohol interventions for adolescents also demonstrate significantly greater reductions in consumption and harm among ‘high-risk’ drinkers; however changes in risk status at follow-up for non-drinkers or low-risk drinkers have not been assessed in controlled trials of brief alcohol interventions.

**Design and methods:**

The study design comprises two linked randomised controlled trials to evaluate the effectiveness and cost-effectiveness of two intervention strategies compared with screening alone. One trial will focus on high-risk adolescent drinkers attending Emergency Departments (Eds) and the other will focus on those identified as low-risk drinkers or abstinent from alcohol but attending the same ED.

Our primary (null) hypothesis is similar for both trials: Personalised Feedback and Brief Advice (PFBA) and Personalised Feedback plus electronic Brief Intervention (eBI) are no more effective than screening alone in alcohol consumed at 12 months after randomisation as measured by the Time-Line Follow-Back 28-day version. Our secondary (null) hypothesis relating to economics states that PFBA and eBI are no more cost-effective than screening alone.

In total 1,500 participants will be recruited into the trials, 750 high-risk drinkers and 750 low-risk drinkers or abstainers. Participants will be randomised with equal probability, stratified by centre, to either a screening only control group or one of the two interventions: single session of PFBA or eBI. All participants will be eligible to receive treatment as usual in addition to any trial intervention. Individual participants will be followed up at 6 and 12 months after randomisation.

**Discussion:**

The protocol represents an ambitious innovative programme of work addressing alcohol use in the adolescent population.

**Trial registration:**

ISRCTN45300218. Registered 5th July 2014.

## Background

Alcohol is a major global threat to public health [1]. Although the main burden of chronic alcohol-related disease is in adults, its foundations often lie in adolescence [2]. A recent survey of alcohol use among 14–15 year olds across 36 European countries found that 87% reported lifetime alcohol use, with 57% consuming alcohol on one or more occasion in the previous month [3]. While the proportion of young people in England aged 11–15 years who reported that they have drunk alcohol decreased from 62% to 45% between 1988 and 2011, the mean amount consumed approximately doubled (from 6.4 to 10.4 units of alcohol per week) between 1994 and 2011 [4]. In England there is a rapid increase in alcohol consumption during school years, with 1% of those aged 11 years reporting weekly alcohol consumption compared with 28% of those aged 15 years [4]. Adolescents in the UK are now amongst the heaviest drinkers in Europe [5].

Alcohol consumption and related harm increase steeply from the age of 12 until 20 years [6]. In middle adolescence (ages 15–17 years) binge drinking (single occasion consumption leading to intoxication) often emerges. Binge drinking is associated with increased risk of unprotected or regretted sexual activity [3,5,7], criminal and disorderly behaviour [5,8], suicide and deliberate self-harm [9], injury [5], drink-driving or allowing oneself to be carried by a drinking driver [10], alcohol poisoning [11] and accidental death [12].

Over the past 15 years the World Health Organisation (WHO), the US Surgeon General, the American Medical Association, and the American Academy of Paediatrics have called for practitioners to carry out screening and brief interventions (SBI) for adolescent drinkers [13-16]. The alcohol strategies in both England and Scotland identify adolescents as a key target group in whom to reduce alcohol consumption and related harm [8,17]. However, while there has been an increase in SBI activity in relation to adults presenting to health care providers, adolescents remain a neglected group.

The term Brief Intervention (BI) encompasses a range of therapeutic processes from advice to extended counselling, and typically is delivered in short sessions on one or more occasion. A number of trials focusing upon young people (aged 12 – 21) have reported significant positive effects of brief interventions on a range of alcohol consumption measures [18-23]. Our recent review of reviews suggests that internet and smartphone delivered BIs (eBI) can significantly reduce alcohol consumption compared with minimal or no intervention controls, and have the added advantage of being more acceptable and easier to implement than more traditional face-to-face interventions [24,25]. Our recently completed study of the prevalence of risky drinking among an adolescent population (aged 10–17 years) found that about 1 in 4 young people presenting to Emergency Departments (EDs) were consuming three or more drinks on one or more occasion over the preceding month, and that this level of consumption was associated with increased physical, social and educational adverse consequences. We also observed a steep transition in drinking prevalence between 13 and 16 years of age.

Several school-based interventions that target non-drinking youths have been found to delay the onset of drinking behaviours [26] and a recent study of adolescents found lower rates of substance misuse initiation among those exposed to a web-based intervention [27]. Web-based alcohol interventions for adolescents also demonstrated significantly greater reductions in consumption and harm among ‘high-risk’ drinkers [28]; however changes in risk status at follow up for non-drinkers or low-risk drinkers have not been assessed in any well controlled trials of BI. Therefore our proposed work will assess both primary outcomes (delayed onset) and secondary outcomes (reduction of consumption and associated harms in those already drinking at a high level) among a population of adolescents.

Recruitment of both ‘high-risk’ and ‘low-risk’ drinkers will have the additional benefit of addressing a major concern among both young people and parents that participation in a trial of this nature may suggest that the participant is drinking at a level that warrants concern and intervention. Young people interviewed as part of this process indicated that they would prefer to take part in a trial if there was no implication that they had an “alcohol problem” and were assured that information about their drinking would not be disclosed to parents or healthcare staff.

Thus we shall conduct two linked trials that will include both high-risk and low-risk drinkers and abstainers, informing them that the study seeks to prevent alcohol-related harm in young people. In addition embedded within the proposed study is an internal feasibility study conducted prior to proceeding to the main trial.

### Objectives of the study

Two linked randomised controlled trials will be conducted. Both trials will evaluate the effectiveness and cost-effectiveness of BI intervention strategies compared with screening alone. One trial will focus on high-risk adolescent drinkers attending Emergency Departments and the other will focus on those identified as low-risk or abstinent from alcohol. In both trials our primary outcome measure will be quantity of alcohol consumed at 12 months after randomisation.

Secondary objectives for each study include:

- To identify key predictors of recruitment to the trials.
- To explore the process of treatment through key psychological constructs that may lead to further refinement of the proposed interventions.
- To identify prognostic factors that improve outcome.
- To explore interactions between participant factors, setting factors, treatment allocation and outcomes.

Our primary (null) hypothesis is similar for both trials: Personalised Feedback and Brief Advice (PFBA) and Personalised Feedback plus electronic Brief Intervention (eBI) are no more effective than screening alone in reducing alcohol consumed at 12 months after randomisation measured by the Time-Line Follow-Back 28-day version. Our secondary (null) hypothesis relating to economics states that PFBA and eBI are no more cost-effective than screening alone.

## Methods

**Figure 1:**
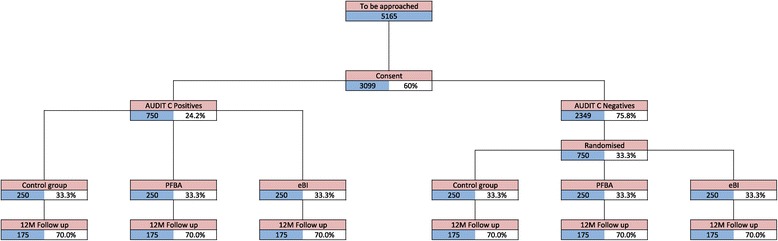
**Trials flow chart.**

The linked trials have been granted ethical approval by the National Research Ethics Service London – Fulham (ref: 14/LO/0721). The linked trials comply with the Declaration of Helsinki and Good Clinical Practice. Full flow diagrams for the studies are shown in Figure 1.

Both trials begin with a randomised internal pilot study to check that the process of recruitment, screening and intervention is effective and does not adversely affect the ED environment. If that pilot is successful, two linked prospective individually randomised controlled trials will be conducted using a similar design, measures and interventions with 6 and 12 months follow up.

### Study settings and participants

The trial will be carried out in 10 EDs across three regions of England: North East, Yorkshire and Humberside, and London. Data collection will be carried out from 10 am to 10 pm seven days per week over a six-month period. During these hours of screening consecutive ED attenders between their 14th and 18th birthdays who meet the inclusion criteria but none of the exclusion criteria will be approached by a researcher and invited to participate in the study once cleared by ED staff to do so.

### Eligibility criteria

Inclusion and exclusion criteria have been chosen to maintain a balance between ensuring the sample is representative of the ED population whilst able to engage with both the relevant interventions and follow up.

Inclusion criteria for participants: aged between 14 and 17 years; alert and orientated; able to speak English sufficiently well to complete the research assessment; resident within 20 miles of the ED; able and willing to provide informed consent to screening, intervention and follow-up; if under 16 years, are ‘Gillick competent’ or have a parent or guardian able and willing to provide informed consent; and own a smartphone or alternatively having access to the internet at home.

Exclusion criteria for participants: severe injury; suffering from serious mental health problem; gross intoxication; specialist services involved because of social or psychological needs; receiving treatment for an alcohol or substance use disorder within the past 6 months; or current participation in other alcohol-related research.

Those who meet the inclusion criteria, fail no exclusion criterion, and score 3 or more on the screening questionnaire – Alcohol Use Disorders Identification Test: Consumption (AUDIT-C) – are eligible for the high-risk study; those who score less are eligible for the low-risk study.

### Consent procedure

The study will be introduced to patients, and their parents or guardians if they are aged less than 16 years, as a study about alcohol, lifestyle and health with the focus on preventing alcohol-related harm in all young people attending ED irrespective of their alcohol consumption. Patients attending ED without their parent or guardian will also be approached to take part if ED staff have confirmed that they are ‘Gillick competent’.

The study will be first introduced by ED staff and then explained in more detail by research staff orally and in writing using the Patient Information Sheet (PIS). If the patient is under the age of 16 years and accompanied by a parent or guardian, the parent or guardian will also receive the PIS. Patients, and parents or guardians if applicable, will have up to 4 hours to ask any questions about the study and to decide whether or not to take part. To obtain the most valid self­report data patients will be told as part of the informed consent procedure that their answers, including alcohol consumption, will not be disclosed to their parent or guardian or the ED staff without their consent.

If patients agree to participate, informed consent will be collected using an electronic device (iPad) overseen by a research assistant who will also introduce and deliver the allocated intervention to each patient in a private area of the ED. Consent to participate will include permission to give the patient's data and contact details to the research staff, and provide the research team with access to the patient’s ED records, and to participate in follow up 6 and 12 months after recruitment.

### Screening and baseline assessment

After consent has been given, by parent or guardian if appropriate, the participant will complete a screening and baseline assessment. All participants scoring 3 or more on the AUDIT-C questionnaire (high-risk drinkers) will be randomised between three groups – two intervention groups and the control group receiving screening alone. Of those scoring less than 3 on the AUDIT-C (low-risk drinkers or abstainers) one in three will be randomly selected to continue with the study and then randomised between three analogous groups. Participants who score less than 3 but are not selected for the study will be thanked for their participation, given a £5 voucher and returned to the care of ED staff.

The screening and baseline assessment includes demographic information and contact details, health and lifestyle questions, the AUDIT-C [29], questions 19, 21 and 22 from the European School survey Project on Alcohol and other Drugs (ESPAD) [5], the Strengths and Difficulties Questionnaire [30], the EQ-5D-5 L [31], and a short service use questionnaire [32]. This takes approximately 10 minutes to complete.

To simplify and enhance data collection we have developed an electronic interface which will only show relevant questions to participants. To maximise completion rates this interface uses an attractive graphic interface. Participants can skip questions or withdraw consent at any stage of the process. All of the instruments have been designed and validated for those aged 14 to 17. The screening and baseline assessment will be conducted by trained researchers with experience of working with adolescents; all researchers will have completed enhanced Disclosure and Barring Service checks in advance. All information given by participants will be treated in confidence.

In total 1,500 participants will be recruited into the trials, 750 high-risk alcohol users and 750 low-risk alcohol users. Participants will be randomized with equal probability, stratified by centre, between a screening only control group and one of the two interventions – a single session of face­to­face Personalised Feedback and Brief Advice (PFBA) or Personalised Feedback plus a smartphone or web-based brief intervention (eBI). All participants will be eligible to receive treatment as usual in addition to any trial intervention.

### Planned interventions

#### Screening only group – Treatment as Usual

After completing the baseline assessment, participants in the screening arm will be thanked for their participation, reminded that a member of the research team will contact them in six and twelve months time to conduct a follow-up interview, and returned to the care of ED staff for usual care.

#### Personalised Feedback and Brief Advice (PFBA)

The PFBA intervention is structured brief advice that will take approximately 5 minutes to deliver. It is based upon an advice leaflet adapted for the target age group in this study from the SIPS Brief Advice About Alcohol Risk intervention [33,34]. It will be conveyed orally to the participant, and tailored to their risk status – high or low. The advice will cover recommended levels of alcohol consumption for young people; summarise the screening results and their meaning; provide normative comparison information on prevalence rates of high and low risk drinking in young people; summarise the risks of drinking and highlight the benefits of stopping or reducing alcohol consumption; outline strategies that they might employ to help stop or reduce alcohol consumption; and highlight goals they might wish to consider; indicate where to obtain further help if they are unsuccessful or need more support. The participant will receive a copy of the leaflet, which includes additional information about alcohol intoxication, alcohol poisoning, and alcohol and the law.

#### Personalised Feedback plus a smartphone or web based brief intervention (eBI)

The eBI smartphone intervention is an offline ­capable mobile web application that work on a variety of platforms but has been optimised for recent iPhone and Android phones. It has been developed using the concept of gamification so users can navigate, explore, learn facts and figures about alcohol, receive personalised feedback and set goals in an engaging format. Its content will adapt to provide the most pertinent information and advice for high-risk or low-risk drinkers. Games components within the web application will support high-risk drinkers to reduce or stop their alcohol consumption, and low-risk users to maintain abstinence or low risk drinking.

For participants without access to a smartphone but with access to the internet through other computerised devices, access to a web based version of the application will be provided with appropriate instructions on its use.

All participants after receiving their allocated intervention (including the screening only group) will be thanked for their participation, reminded that a member of the research team will contact them in six and twelve months time to conduct a follow-up interview, receive a £5 voucher and returned to the care of ED staff.

## Fidelity

To assess the fidelity of the delivery of the interventions to the norm we shall record a random sample of 20% of the interventions delivered by each researcher using the iPad application. The resulting audio files will not contain personal identifiable data and will be stored separately from the patient data.

## Follow up assessments

All participants will be followed up at 6 months after randomisation with a brief set of questions and then at 12 months for a full assessment. Follow up interviews will be conducted over the telephone, face ­to ­face or electro-nically via web survey, as preferred by the participant – by research assistants trained in the administration of the assessment tools and blinded to the group allocation of the participants. Letters of thanks will be sent to participants after each follow up stage. On completion of each follow-up interview participants will receive a gift token for £5 in recognition of their participation.

## Outcome measures

### Primary outcome measure

Total alcohol consumed in standard UK units (1 unit = 8 g ethanol) over the 28 days before the 12-month follow-up, assessed using the Timeline Follow Back interview 28-day version (TLFB28) will be our primary outcome measure. TLFB28 is an established valid and reliable method of ascertaining alcohol consumption in adolescent populations over a reference period ranging from 7 to 365 days [35].

### Secondary outcome measures

The TLFB28 will also provide secondary outcomes of percentage of days abstinent, drinks per drinking day, and number of days of heavy episodic alcohol use. Participants will also be asked questions about alcohol use over their lifetimes and the past year, and the consequences of alcohol consumption using questions 19, 21, and 22 from the ESPAD study [5] at baseline. Hazardous alcohol use will be assessed using the extended AUDIT-C questionnaire [29] at baseline and after 6 and 12 months. General health and functioning will be measured using the Strengths and Difficulties Questionnaire [30] at baseline and 12 months. We shall also ask about service use including previous use of health and social services, school attendance and contact with criminal justice; and health utility (EQ-5D-5 L; 31) at baseline, 6 and 12 months.

### Process outcome measures

Expectancy will be measured using the ESPAD Question 21 [5] at baseline and 12 months after randomisation. Adherence to the eBI intervention will be assessed by monitoring remotely either when the smartphone device is connected to the internet, or else access to the web application. To assess treatment fidelity a random sample of treatment sessions will be audio-recorded, stratified by researcher, intervention, risk group and centre. These will be assessed for fidelity to behavioural change components using a behaviour rating scale employed in previous studies of alcohol brief intervention. Assessment will be conducted by two independent expert clinician assessors and analysed to take account of agreement between them.

### Economic outcome measures

The primary outcome measure for the economic evaluation in the trial will be the quality adjusted life year (QALY) as assessed by the EQ-5D-5 L [31]. The secondary economic outcome measure will be alcohol consumption – derived from the TLFB28 for the high-risk group and from the maintainence of low-risk consumption for the low-risk group. The study will also collect data on the costs of the interventions and on the use of NHS, social care, criminal justice and other resources over 12 months follow-up period, using a bespoke version of the Client Service Receipt Inventory (CSRI).

## Analysis

### Sample size calculation

For both studies the sample size addresses the effect of interventions on the primary outcome measure – alcohol consumption at 12 months after randomisation. To detect the ‘clinically important’ effect size of 0.3 (that previously used for adults) with a significance level of 5% and statistical power of 80% when using a two-sided continuity-corrected test will require 175 in each of the 3 groups, yielding a target of 525 analysable participants in each of the two trials.

Predicting that follow-up at 12 months will be 70% we need to randomise 750 high-risk drinkers and 750 low-risk drinkers. Based on the estimated prevalence of 24.2% for high-risk drinking (namely AUDIT-C ≥ 3) from our earlier survey and a consent rate of 60%, we would need to approach 5165 potential participants over the recruitment period. Of these our survey predicts that 2,350 will be low-risk drinkers consenting to the study. Therefore we shall initially sample one third of these to participate in the study; and we shall adjust this ratio if necessary.

To assess fidelity we plan to record a random sample of 20% of allocations, stratified by researcher, intervention, risk group, and centre. This will provide a total sample of 300 – 50 in each of the six intervention groups across for high and low risks.

### Statistical analysis

We shall review our basic design at the end of the internal pilot study, when we have recruited 100 participants, to assess recruitment, consenting and adherence. We shall study the impact of the research on observed screening and prevalence rates to confirm our design, especially the number of centres needed in the main trial.

The outcomes for both studies will be analysed in a similar manner. The primary analysis will be by treatment allocated using a two-sided 5% significance level. Analysis and results will be presented in accordance with the CONSORT guidelines. The primary outcome is total units of alcohol consumed in the 28-days before the 12-month follow-up. After checking the distributional assumption of normality, and transforming the data if necessary, we shall conduct multivariate analysis of covariance adjusting for baseline AUDIT-C score, age, gender and centre to estimate the magnitude of differences between groups. Analysis will be presented as mean differences between groups with 95% confidence intervals. Multiple imputation will be employed with sensitivity analysis to adjust for missing data. Secondary outcomes will be analysed in a similar manner.

Analysis will also model the relationship between pre-randomisation factors and observed outcomes, including whether positive or negative at 12 months according to AUDIT-C. This will take the form of a linear or logistic regression including interaction terms for allocated group. These models will also assess the effect of adherence and fidelity of interventions on observed outcomes.

### Cost-effectiveness analysis

The clinical effectiveness analysis will be complemented by an economic evaluation that will estimate the cost-effectiveness of each intervention versus screening alone. Alcohol use disorders and associated problems generate high costs in health care and in society more widely, including costs to the criminal justice and social care systems. The incremental cost effectiveness of the two interventions versus screening will thus be assessed from a societal perspective that will account for resource savings beyond the NHS.

The costs of screening and of delivering the two interventions will be estimated by prospectively monitoring resource inputs to each arm of the trial, including training, valued using standard methods [36]. Effects on NHS and non-NHS costs will be estimated from information gathered on patient contact with primary care, secondary care, specialist health services, social services and criminal justice. These will be assessed retrospectively using the CSRI tool. Service use will be valued using local costs where possible supplemented by national sources and information from previous alcohol studies [34,37,38].

Cost effectiveness will be assessed in terms of the incremental net health benefit (NHB), which converts costs and Quality Adjusted Life Years (QALYs) gained, as estimated from EQ-5D-5 L scores and UK population utility values, into a single monetary value. Differences in QALYs will be estimated from the ‘area under the utility curve’. Both one-way and multi-way sensitivity analyses will be carried out to explore our basic assumptions. Non-parametric bootstrapping will be used to investigate joint uncertainty in costs and effects via cost effectiveness acceptability curves.

Three secondary analyses will be undertaken. The first will adopt a narrower NHS perspective including only costs and savings relating to the NHS and personal social services. This will us to compare the NHB with other interventions assessed using the narrower perspective recommended by the National Institute for Health and Care Excellence (NICE) for the economic evaluation of NHS interventions. The second will compare total societal costs with decreased alcohol consumption for the high-risk group. The third will compare total societal costs with the proportion of the low-risk group maintaining moderate alcohol use.

### Qualitative analysis

To explore acceptability of trial tools and processes to young people presenting at emergency departments we shall use semi-structured interviews to study: issues relating to consent by young people aged 14–17 years; alcohol screening; the baseline questionnaire and the burden on emergency care; and young person experiences of intervention delivery. A purposive sample of participants will be selected from the pool of participants in the two linked trials for interviews about the experience and acceptability of receiving the interventions. The sampling will cover the key variables of interest including: gender; ethnicity; age; level of alcohol use; area of the country (North East, Yorkshire and Humber or London), allocated intervention and whether it was delivered. To achieve this, a sample of at least 20 young people is likely to be required. However, recruitment will continue until data saturation is deemed to have been achieved, that is no new issues or themes are arising in the interviews.

A participant information leaflet explaining the qualitative study will be sent to a sample of young people who provided consent to be contacted for interview and, when appropriate, their parents. The young people will be invited to attend a one-to-one semi-structured interview. The interviews will be conducted in a venue that is appropriate, taking consideration of confidentiality, risk assessment, participant convenience and comfort. The purpose of the interview will be explained orally to the participant before arranging interview time and date. Interviews will be audio-recorded and transcribed to support data analysis. Though the topic guide will be produced before the start of interviews, and state the aims and objectives of the study, emergent issues will be explored as they arise.

Data will be subjected to framework analysis, which is appropriate for qualitative health research with objectives linked to quantitative investigation. This analytic strategy is characterised by an approach more deductive than inductive, in which analysis is structured around given themes so findings have detailed relevance to applied research questions. Based on interview notes and review of the interview recordings, important or recurrent themes in interviewees’ responses will be identified. These will be combined with a list of key themes of research interest derived from the aims and objectives of the study. All transcripts will be repeatedly read and coded within this framework of prior headings. Data coded under each heading will be reviewed to produce a detailed description, and revised through the course of analysis to take account of all material under that heading. Finally the descriptions of headings within the framework will be compared and the relationships between them elaborated to provide a consistent interpretation of the dataset as a whole. Analysis will continue throughout the process of data collection, and will be discussed within the subgroup of the research team tasked with managing the qualitative study. This analytic process will assist in the identification of emerging themes and enhance credibility of the thematic framework and interpretation.

## Discussion

This protocol represents an innovative and ambitious programme of work evaluating the use of novel interventions to address alcohol use in adolescent populations. The study addresses both effectiveness and cost-effectiveness and is designed to provide evidence of what works in reducing at-risk drinking in this population in addition to how to reduce the progression from low to high risk drinking.

## Ethics and confidentiality

The study has been granted ethical approval by the National Research Ethics Service London – Fulham (Ref num 14/LO/0721). There are no anticipated risks in relation to either treatment. There is no documented evidence of adverse events arising from any of the proposed interventions.

All trial data will be identified using a unique trial identification number. No personally identifiable information will be held beyond the final 12-month follow up. Analytical datasets will not contain any patient identifiable information. Anonymised data will be retained for a period of 60 months.
